# Supporting migrant groups to reduce tobacco-related harms and create smoke-free family environments: Future priorities and research gaps

**DOI:** 10.18332/tid/189356

**Published:** 2024-06-13

**Authors:** Rachel O’Donnell, Olena Tigova, Piotr Teodorowski, Nazmy Villarroel-Williams, Anzhelika Shevchuk, Olena Nesterova, Yuliia Arabska, Alban Ylli, Gentiana Qirjako, Esteve Fernández, Sean Semple

**Affiliations:** 1 Institute for Social Marketing and Health, Faculty of Health Sciences and Sport, University of Stirling, Stirling, United Kingdom; 2 World Health Organization Collaborating Center on Tobacco Control, Catalan Institute of Oncology (ICO), L'Hospitalet de Llobregat, Barcelona, Spain; 3 CIBER of Respiratory Diseases (CIBERES), Madrid, Spain; 4 Tobacco Control Research Group, Bellvitge Biomedical Research Institute (IDIBELL), L'Hospitalet de Llobregat, Barcelona, Spain; 5 School of Medicine and Health Sciences, University of Barcelona, L'Hospitalet de Llobregat, Barcelona, Spain; 6 Faculty of Health Sciences and Sport, University of Stirling, Stirling, United Kingdom; 7 Department of Psychology, Sociology and Politics, Sheffield Hallam University, Sheffield, United Kingdom; 8 Public Health Center of the Ministry of Health of Ukraine, Kyiv, Ukraine; 9 Faculty of Medicine, University of Medicine Tirana, Tirana, Albania

**Keywords:** migrant health, smoke-free home, secondhand smoke, smoking cessation

There are approximately 1 billion migrants globally, about 1 in 8 of the global population. These include 281 million international migrants and 82.4 million forcibly displaced (48 million internally displaced, 26.4 million refugees, and 4.1 million asylum seekers)^1^. Europe presently serves as the primary destination for international migrants, accommodating 87 million individuals, representing 30.9% of the international migrant population^2^. Despite the right to health for everyone expressly set out in various international treaties and case law^3^, a discrepancy exists between emphasis on health rights and equity, and the actual provision of healthcare and services for migrants, refugees, and asylum seekers. To address this gap, the World Health Organization has recently published its first global research agenda on health and migration^4^ to guide research efforts and shape policy and practice. Priorities include research on developing interventions to improve service provision, quantifying the burden of non-communicable diseases (NCDs) among migrant and refugee groups, and research on how health and disease are conceptualized and expressed differently by people from different cultural backgrounds. Building on a recent two-day workshop hosted by the University of Stirling, this Editorial discusses these priorities concerning the health harms caused by smoking among migrant populations with a particular focus on how to increase the provision of smoke-free family environments and protect children and non-smokers from the effects of secondhand tobacco smoke (SHS).

## Quantification of the burden of NCDs among migrant and refugee groups

More than 8 million people die prematurely every year from tobacco use – more than 7 million of those deaths result from direct tobacco use and around 1.3 million deaths are related to non-smokers who are exposed to SHS^5^. A total of 508 million children under 15 years of age were exposed to SHS at home across just 21 countries studied in 2015^6^. There is no safe level of exposure to SHS, which increases the risk of many childhood illnesses including asthma, croup and bronchiolitis^7^.

The limited studies conducted on smoking-related disease and mortality among migrant groups are outdated and provide contradictory results, given the nature of migration, with studies conducted in different locations and focusing on different migrant groups^8,9^. For example, one study suggests that higher tobacco use places migrants at a higher risk for cardiovascular disease and other health problems^9^, while another suggests low smoking-related mortality is a significant ‘mortality advantage’ for migrant groups in the US^8^. Data on SHS exposure in the home may not be captured.

Data on healthcare utilization by migrant groups are variable, which makes cross-country comparisons difficult and even inadequate in some instances^10^. Many countries do not record migration information within health records, and all use disparate criteria to classify migrant status. The lack of comparative data hinders public health surveillance^10^. Current migration drivers include worldwide economic instability, exacerbated by the COVID-19 pandemic, and conflicts, including wars^11,12^. Europe has experienced an influx of Ukrainian refugees seeking temporary protection – more than 4.2 million Ukrainians, predominantly women and children – with the highest number settling in Germany, Poland, and the Czech Republic. Preliminary evidence from Poland indicates that most Ukrainian migrants are in good health, with many having a medium or high socioeconomic status^13^. However, there is a lack of evidence regarding behavioral risk factors, including tobacco use and SHS exposure among Ukrainian migrants, and how high levels of psychological distress impact and alter health behaviours^14^. Environmental damage associated with climate change is expected to increase the number of asylum seekers entering Europe and elsewhere in the future. Ageing European populations and the need for migrant workers will continue to be another important driver. Increasing migration numbers are likely to be a long-term phenomenon, and all countries must work to develop effective data collection systems to inform effective public health surveillance.

## The development of public health interventions targeting migrant populations

Delivering smoking cessation and tobacco-harm reduction services that are appropriate for migrants is challenging as these communities are not homogenous, and a ‘one-size-fits-all approach’ might not be effective^15^. For example, the stress induced by forced migration as a result of persecution, war, conflict, and violence, and/or poor living and working conditions, may increase the likelihood of addiction and smoking^16^. Some migrants come from countries with higher smoking rates. For example, a few studies have identified a high prevalence of smoking and exposure to SHS in migrants from Eastern European countries^17,18^. This may be due to lifestyle and cultural practices, and the ability to change home smoking behaviors may be related to age at migration, socioeconomic status, housing, community characteristics, language ability, or other unknown determinants. Many migrants also come from countries with different legal frameworks for tobacco control and different cultural and social approaches to tobacco use behavior, knowledge and attitudes.

The limited available evidence suggests that services need to better adapt to migrant community needs and expectations once they settle^18,19^, and better understand cultural values related to smoking^19^. The dynamic acculturation process – where immigrants’ behaviors change due to interactions with individuals in their (new) social and cultural environment – also has implications. A recent systematic review^20^ found more acculturated women (non-Western by birth, migrating to a Western host country) were more likely to smoke than less acculturated women, and the contrary was observed among men. This suggests that migrant health promotion programs may benefit from employing gender-specific strategies.

There is a lack of adapted intervention programs for migrants who wish to stop smoking, potentially due to challenges reaching migrant populations, financial costs, and language barriers^21^. Research suggests that immigrants have a lower likelihood of being prescribed nicotine replacement therapy (NRT), which may be explained by health insurance status and/or communication challenges between immigrant patients and health professionals^19,22^. Further research is warranted to understand this disparity given that NRT products are commonly used as a quit smoking medication and, more recently, as a means of temporary abstinence from smoking in the home^23,24^ or during hospitalization^25^. While there is increasing interest in studying the impact of parental migration on adolescents’ SHS exposure in their homes^17^, tailored interventions to support the adoption of a smoke-free home within migrant groups are also sparse, and likely required.

## Research on how health and disease are conceptualized and expressed differently by people from different cultural backgrounds

There is little published research examining migrants’ thoughts and feelings about the harms associated with cigarette smoking and SHS exposure, nor whether risk perceptions change as a result of migration to a country where social norms associated with smoking differ. A few studies conducted with migrant groups in China suggest that non-smokers’ knowledge of smoking-related risks is significantly higher than that of individuals who smoke, indicating that health education might help to reduce smoking prevalence and reduce the likelihood of smoking initiation^26^. One study reporting on barriers to accessing and participating in smoking cessation services suggests working hours, low confidence in cessation services, language barriers, cultural barriers, and unsuitability of services for Chinese migrants living in Glasgow^19^. An adapted intervention program for Turkish-speaking migrants who want to quit smoking in Switzerland has shown initial promise^21^.

We are unaware of any published research that explores knowledge, beliefs, and behaviors related to SHS exposure and smoking in the home in migrant groups. This is important because inaccurate beliefs and/or incomplete knowledge about SHS can contribute to exposure among other household members^27^. An enhanced understanding of attitudes and knowledge would also be beneficial for informing the development of future interventions, alongside identifying the individual, household-level, and structural barriers (and opportunities) that might hinder (or help) creating and maintaining a smoke-free home. For example, migrants often experience frequent moves between temporary accommodations, likely impacting their feelings of ‘control’ over their home environment. Some hostel and hotel accommodations may have smoking rules that mean smoking is not permitted; others may have little enforcement and high degrees of tobacco smoke incursion from neighbors. It is not difficult to envisage that smelling smoke from neighboring apartments makes implementing a smoke-free living space harder. High-density housing arrangements for migrants may make it particularly difficult to protect family members from SHS. The language used to explain the concepts around ‘smoke-free homes’ may introduce further complexity. ‘Smoke-free’ may be interpreted as a home free of smokers (i.e. a home where no one living there smokes) rather than the public health definition of a place where smoking is not permitted. Similarly, there may be issues about what constitutes the ‘home’ space, with some smokers considering hallways, entrances, bathrooms, verandas, and balconies not included in the definition. There are likely to be differences in ‘smoke-free home’ understanding between migrant groups depending on local terminology, how well the phrase ‘back-translates’ to the origin country’s language, and how common the concept was in the community from where the migrant previously lived.

Language barriers present a key challenge in working with migrant communities. Researchers should ensure that appropriate resources are available for translators and interpreters where required. Key persons from migrant communities should be involved in implementing strategies in their communities, settings, and networks^20^. The importance of public involvement in increasing the relevance and applicability of public health research to end-users is now widely recognized, and involving representatives from migrant groups as public contributors has the potential to transform research. Time and resources are required to do this effectively, to build relationships and trust, and to ensure accessibility and transparency^28^. Strategies for developing culturally sensitive, smoking behavior-change interventions – whether focused on cessation or creating a smoke-free home – require establishing robust community partnerships, conducting formative research, and pilot testing with the target population^29^.

## Conclusion

As more countries implement or move towards implementing endgame strategies for tobacco control^30,31^, it is imperative that future work includes a clear focus on qualitative and quantitative research by and with members of communities most affected by smoking, including migrant groups. Research to inform the development of effective interventions and health services to increase knowledge, aid smoking cessation, and provide children and non-smokers with a smoke-free home environment is key to reducing tobacco-related harms and helping improve the health of migrant populations.

## Data Availability

The data supporting this research are available from the authors on reasonable request.
